# Research Progress of Epigenetic Modifications in Myopia

**DOI:** 10.7150/ijms.110640

**Published:** 2025-06-23

**Authors:** Yinqiao Zhang, Zhaohui Yang, Miao Zhang, Yuanting Yang, Zhongyu Ma, Mengke Wu, Hongsheng Bi, Dadong Guo

**Affiliations:** 1 Shandong University of Traditional Chinese Medicine, Jinan 250002, China.; 2 Affiliated Eye Hospital of Shandong University of Traditional Chinese Medicine, Jinan 250002, China.; 3 Medical College of Optometry and Ophthalmology, Shandong University of Traditional Chinese Medicine, Jinan 250002, China.; 4 Shandong Provincial Key Laboratory of Integrated Traditional Chinese and Western Medicine for Prevention and Therapy of Ocular Diseases; Shandong Academy of Eye Disease Prevention and Therapy, Jinan 250002, China.

**Keywords:** myopia, epigenetic modification, DNA methylation, RNA methylation, non-coding RNA, histone modification

## Abstract

Myopia, also known as nearsightedness, refers to a refractive error of the eye that causes parallel rays of light to focus in front of the retina, affecting distance vision. High myopia significantly increases the risk of pathological myopia, leading to severe complications and an increased likelihood of myopia-related eye diseases. In recent decades, the incidence of myopia has continued to rise, posing significant social and human health issues. The complex interplay between genetic and environmental variables affects the development of myopia. Gene control depends to a large extent on epigenetic changes, which are reversible, inheritable, and sensitive to ecological shifts. Therefore, the pathophysiology and development of myopia are tightly linked to gene regulation mediated by epigenetic changes. To explore epigenetic modifications related to myopia, a PubMed search was conducted using keywords such as epigenetic modification, epigenetics, DNA methylation, RNA methylation, non-coding RNA, long non-coding RNA, short interfering RNA, microRNA, ribosomal RNA, circular RNA, transfer RNA, histone modification, histone methylation, and histone acetylation. This review presents the current understanding of these epigenetic modifications in myopia to provide new insights for advancing myopia research.

## 1. Introduction

Myopia is a spherical equivalent ≤ -0.5 diopter refractive error 1. Myopia is usually caused by an excessively long eyeball, especially the long vitreous cavity 2. Patients with high myopia (HM) are at a higher risk of various secondary complications, including glaucoma, cataracts, choroidal neovascularization (CNV), optic neuropathy, uveitis, retinal detachment, and myopia-related macular degeneration 3.

In the twenty-first century, myopia has become a severe public health problem 4. Myopia has become far more common than it was in earlier decades. By 2050, 938 million people (9.8% of the world's population) will suffer from HM, while 4.758 billion individuals (49.8% of the world's population) will have myopia. This growing demand will require significant healthcare resources to manage nearly 1 billion HM patients worldwide 5. The incidence of myopia continues to rise, which has a substantial impact on human eye health.

**Figure 1:**
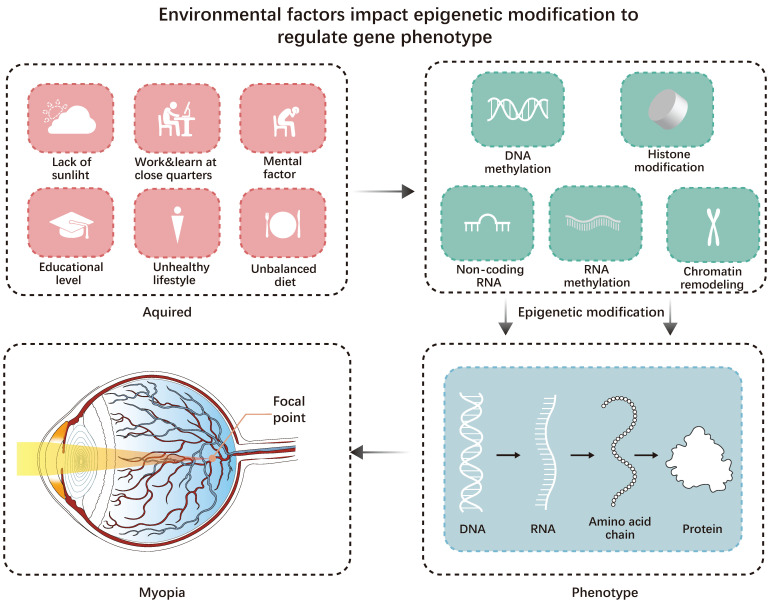
Environmental factors impact epigenetic modification to regulate gene-phenotype. Acquired factors can all affect epigenetic modifications, leading to changes in the genetic phenotype and ultimately triggering myopia.

For most myopic patients, modern lifestyle factors may be the main factor contributing to myopia 1. For instance, increased educational pressure and decreased outdoor time for children are the main lifestyle factors contributing to myopia. However, Inuit communities have much lower educational pressure than developed East and Southeast Asian countries. The lack of outdoor light may have a more significant impact on them 6. Hereditary factors influence an individual's susceptibility to way-of-life risk factors for myopia (Figure 1).

Epigenetics affects many diseases. Some diseases, including autoimmune diseases and cancer, may result from disturbances in the transcription patterns controlled by epigenetic processes 7. Inheritance and external factors may influence disease progression through epigenetic changes.

Diseases related to the eyes are significantly influenced by epigenetic changes, like glaucoma, cataracts, corneal diseases, proliferative vitreoretinopathy (PVR), age-related macular degeneration (AMD), eye tumors, and certain systemic diseases like endocrine and immune disorders (uveitis, diabetic retinopathy, Sjögren's syndrome). For example, Wang *et al.* found that the most significantly associated demethylated region in people with Sjögren's syndrome is located in a gene controlled by type I interferon (IFN1), which is associated with B cell invasion and disease progression disease. Sjögren's syndrome is linked to MX dynamin-like GTPase 1 (MX1), RUNX family transcription factor 2 (RUNX1), and lymphotoxin alpha (LTA) 8. It has also been found that the methylation level of the IL17RC promoter is significantly reduced in the retina, choroid, and peripheral blood of patients with AMD, leading to elevated IL17RC expression and that IL17RC may be a biomarker for AMD diagnosis 9. Myopia is one of the most common eye diseases, and its occurrence and development are significantly affected by epigenetic modifications.

## 2. Molecular Mechanisms of Myopia

### 2.1 Myopia pathogenesis

The combination of environmental and genetic factors causes myopia 10. External factors complete the information in the genome and continuously produce molecular-level information 11. Zhang *et al.* calculated an environmental and genetic index value of 0.125 through a survey of Chinese children aged 6 to 9, suggesting that genetic variables may have an impact of 12.5% on the development of myopia. 87.5% of myopia progress may be influenced by external factors 12.

### 2.2 Heredity and myopia

The appearance of myopia is significantly influenced by genetic markers associated with myopia. Myopia has multiple inheritance mechanisms, including autosomal dominant, autosomal recessive, and X-linked. Autosomal dominant inheritance means that the defective gene is expressed dominantly. As long as one allele of the gene is abnormal, it can lead to autosomal dominant inheritance, and 50% of the children have the possibility of developing the disease. A pathogenic gene inherited in an autosomal recessive manner is located on an autosome, and its trait is recessive, meaning that the disease manifests only in homozygous individuals. Offspring have a 25% probability of inheriting the disease, with an equal likelihood for both male and female children. X-linked inheritance refers to the inheritance pattern of genes located on the X chromosome and can be classified into X-linked recessive and X-linked dominant inheritance. A few rare cases of pathological myopia occur exclusively in males, with female carriers transmitting the condition to the next generation. Li *et al.* discovered a novel SNP (rs1064261). They found that polymorphisms in the mechanistic target of rapamycin kinase (MTOR) and platelet-derived growth factor receptor alpha (PDGFRA) genes were associated with different degrees of myopia severity 13. Zhao *et al.* discovered that the eyes of photoreceptor-deficient mice had lower phosphodiesterase 4B (PDE4B) levels and that PDE4B knockout mice had higher myopic dioptre shift. These findings suggest that PDE4B inhibition and down-regulation cause myopia 14.

## 3. The State of Research on Epigenetic Changes in Myopia

### 3.1 Methylation of DNA

DNA methylation is the most characteristic epigenetic mechanism 15. It refers to the covalent addition of a methyl group to the 5th carbon of cytosine in CpG dinucleotides under the action of DNA methyltransferases (DNMTs), which is essential for the growth of mammals 16,17.

Some studies have found that single-point methylation alterations in NGFI-A binding protein 2 (NAB2), AP-1 transcription factor subunit (FOS), and early growth response 1 (EGR1) are associated with changes in in the speed of optically induced eye development rate, but not with large-scale changes 18. Through DNA methylation, external environmental changes can affect eye growth and are associated with the onset and incidence of myopia.

DNA methylation generally silences genes, while DNA demethylation promotes gene expression. The delicate balance between continuous DNA methylation and demethylation forms the cell's final methylation pattern.

#### 3.1.1 DNA demethylation of myopia-related genes

The gene that promotes myopia can increase its expression by hypomethylating its promoter region. In the guinea pig form deprivation myopia (FDM), Ding *et al.* discovered that hypomethylation of four CpG sites in the promoter region of the insulin-like growth factor 1 (IGF1) gene promotes an increase in IGF1 transcriptional levels in the sclera, which leads to myopia 19. A team in Singapore found that in myopic children, the methylation level of the upstream homoeobox A9 (HOXA9) gene promoter region of the retinal pigment epithelium (RPE) is low, resulting in increased HOXA9 expression, which in turn causes increased expression of insulin-like growth factor 1 receptor (IGF1R), matrix metalloproteinase 2 (MMP2), fibroblast growth factor 2 (FGF2) and transforming growth factor beta (TGFB). These factors are responsible for the growth of the RPE, elongation of the eyeball, and the deepening of myopia 20. Swierkowska's team found that the cytosine-phosphate-guanine sites (CpG islands) in the promoter region of the protocadherin alpha 10 (PCDHA10) gene cluster at the 5q31 locus was most significantly hypomethylated, overlapping with the intron of PCDHA19, which may be connected to the early onset of HM 21.

#### 3.1.2 High levels of DNA methylation in genes linked to myopia

When the methylation level in the promoter region of the myopia suppressor gene is too high, the expression of the gene is inhibited. A study found that the methylation level of long interspersed nucleotide element 1 (LINE-1) in myopic individuals was significantly higher than in the normal group. FDM mice also showed higher methylation of LINE-1 in leukocytes, retina, and sclera. Dopamine (DA) can reduce LINE-1 methylation and DNA methyltransferases (DNMTs), revealing part of the mechanism of DA treatment of myopia 22. Zhu *et al.* found that HM eyeballs undergo early vitreous liquefaction due to excessive axial length (AL), which increases the oxygen content around the lens and subjects myopic eyes to more oxidative stress. Increased expression of DNMT1 induces hypermethylation of antioxidant genes (thioredoxin reductase 2 (TXNRD2) and glutathione S-transferase pi 1 (GSTP1)), inhibiting the expression of antioxidant genes, promoting the development of HM 23. One study found that the high methylation of the CpG islands in the promoter of the collagen type I alpha 1 chain (COL1A1) in the sclera of FDM mice hindered the transcription of COL1A1, thereby inhibiting the synthesis of scleral collagen and accelerating the onset of myopia 24. Compared with individuals with age-related cataracts (ARC), nuclear cataracts and HM patients have more hypermethylation in the CpG regions in the crystallin alpha A (CRYAA) promoter. Furthermore, there is less CRYAA in the lenses of HM patients. The reduced CRYAA expression may make lens proteins more susceptible to oxidative stress associated with aging, which could explain how nuclear cataracts worsen the pathophysiology of HM 25.

Early onset of myopia is also directly linked to high DNA methylation. Seow's team found that the higher frequency of early-stage myopia in 3-year-olds may be related to DNA site-specific CpG methylation in neonatal umbilical cord tissue 26. One type of transcription factor involved in developing the central nervous system and the eye is paired box 6 (PAX6). It is the dominant gene for eye development 27. A study found that grandmothers who smoked during pregnancy could reduce the prevalence of myopia in their grandchildren before seven. Whole genome methylation analysis is consistent with this view, and an association near the PAX6 gene indicates a clear association between methylation and early-onset myopia 28. A study comparing HM cases with typical cases (4 to 12 years old) revealed that myopia-related genes such as suppressor of cytokine signaling 1 (SOCS1), zinc and ring finger 3 (ZNRF3), presenilin 1 (PSEN1), PAX6, adaptor related protein complex 1 subunit beta 1 (AP1B1), adenylate cyclase 3 (ADCY3), the regulator of G protein signaling 5 (RGS5), serum response factor (SRF) and growth factor receptor binding protein 2 (GRB2), are highly methylated, promoting the development of myopia 29. A study found 1541 highly methylated CpGs, including PAX6, which interact with one another and are associated with the growth and maintenance of retinal ganglion cells (RGCs), changes in axon length, synaptic connectivity, and dysfunction of corneal and sclera 30. The methylation level of the PAX6 gene in the myopic group of junior high school students was slightly higher than that in the non-myopic team, and the more severe the myopia, the lower the level of the methylation PAX6 gene. This proves that early myopia is caused by hypermethylation of the PAX6 gene promoter 31. Multiple genes interact to cause myopia, not a single gene.

#### 3.1.3 DNA methylation interacts with non-coding RNAs to promote myopia progression

**Figure 2:**
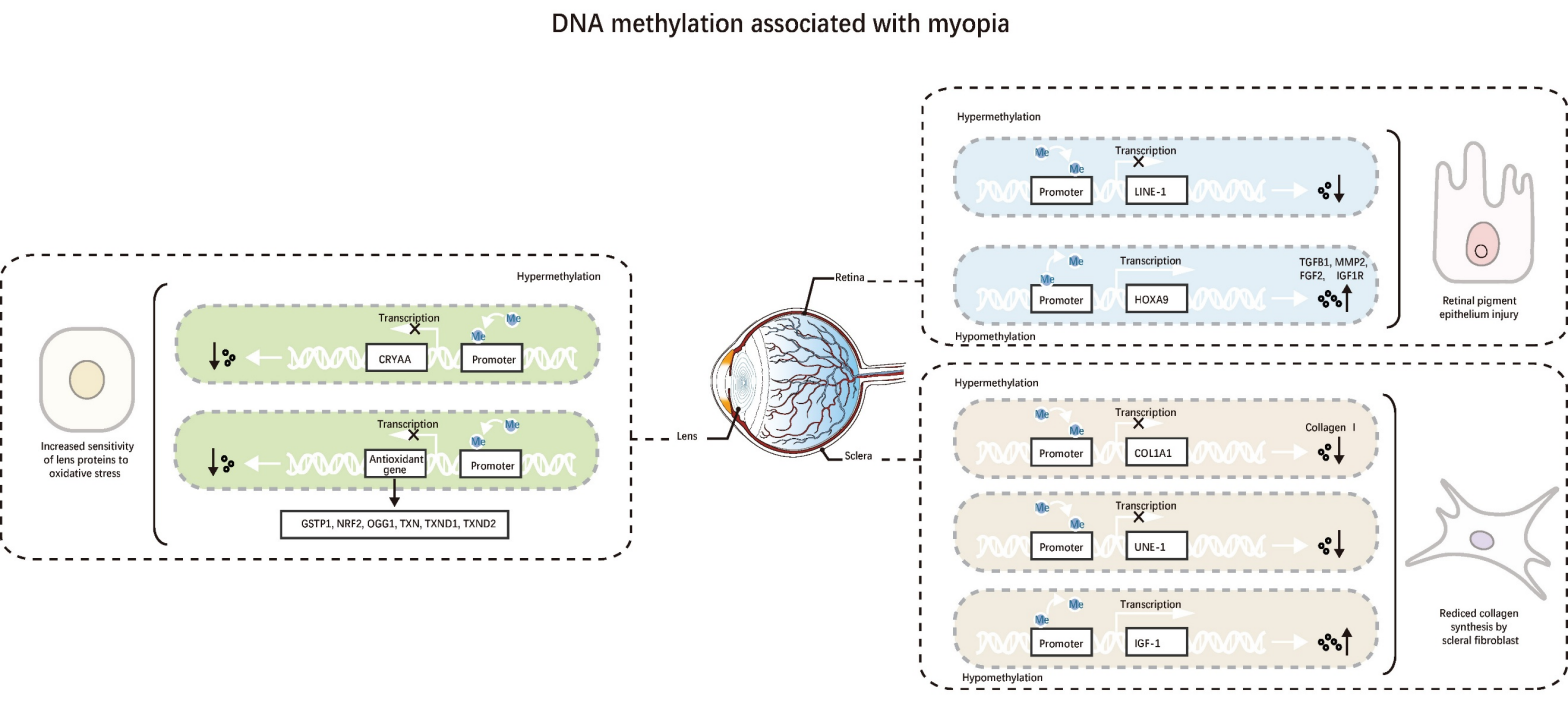
DNA methylation is associated with myopia. 1. DNA methylation related to myopia in the lens increases crystallin's susceptibility to oxidative stress. 2. DNA methylation related to myopia in the retina, ultimately causing damage to RPE. 3. DNA methylation in the sclera associated with myopia, leading to reduced collagen synthesis by scleral fibroblasts.

The genes encoding miR-1178, miR-LET-7A2, miR-885, miR-548-I3, miR-6854, miR-675, miR-LET-7C, and miR-99A have hypomethylated CpG islands in their promoter regions, while the genes encoding miR-3621, miR-34C, and miR-423 have highly methylated CpG islands in their promoter regions. These differences in DNA methylation levels affect the transcriptional regulation of target genes and alter the pathways, leading to HM 32. This study shows that epigenetic modifications of DNA methylation and micro RNAs (miRNAs) do not exist in isolation but are interconnected and interact (Figure 2).

In short, methylation markers show how myopia genes are linked to epigenetic targets, how DNA methylation and non-coding RNA interactions affect eye growth, and how the environment affects gene expression. Methylation targets can prevent myopia from developing, while methylation markers can be used to detect early-onset myopia. However, individual variability and the high cost and complexity of methylation detection make their implementation more challenging. More research is needed to determine the precise dynamic equilibrium mechanism between methylation and demethylation.

### 3.2 Non-coding RNAs

Although non-coding RNAs (ncRNAs) were once considered “junk,” advances in molecular biology have revealed that they control gene expression in various cellular networks and processes. These ncRNAs include regulatory and housekeeping ncRNAs 33,34.

#### 3.2.1 microRNAs

miRNA is a short (less than 22 nt) endogenous ncRNA that regulates gene expression post-transcriptionally through translation inhibition or messenger RNA (mRNA) degradation 35,36. By controlling autophagy, gene expression, and scleral remodeling, choroidal fibrosis, among other processes, miRNAs are known to contribute to the development of myopia 37.

##### miRNAs associated with myopia in aqueous humor

In both HM and normal samples' aqueous humor (AH), Li *et al.* found 37 miRNAs that were differently expressed. Among these is hsa-miR-142-3p, which targets and inhibits TGFB1, suppresses type I collagen expression in human scleral fibroblasts (HSFs), and positively correlates with ocular AL 38. The myopic group's total RNA content was 2.78 times higher than the cataract groups. Fifteen miRNAs are specific to myopia, while four were missing. It has been discovered that six well-known myopia-related genes are targeted by five myopia-specific miRNAs (has-miR-582-3p, has-miR-885-3p, has-miR-19b-3p, has-miR-450b-5p, and has-miR-17-5p): cyclic nucleotide-gated channel subunit beta 3 (CNGB3), lumican (LUM), vascular endothelial growth factor A (VEGFA), cholinergic receptor muscarinic 2 (CHRM2), IGF1, and adenosine A2a receptor (ADORA2A). The CHRM2 may be a target of the missing miRNA in myopia (has-miR-378a-5p) 39. Compared to cataract patients, HM patients have higher expression of LET-7b/c/e, LET-7i, miR-98, miR-103, miR-214, and miR-29b in their AH. These abnormally produced miRNAs may regulate some signaling pathways, including phosphatidylinositol 3 kinase/protein kinase B (PI3K/AKT), hypoxia-inducible factor 1 subunit alpha (HIF1A), mitogen-activated protein kinase (MAPK), and tumor necrosis factor (TNF) 40. Zhu *et al.* discovered that HM patients have noticeably increased levels of miR-29a in AH compared to cataract patients. Although miR-29a has no significant effect on cell proliferation, it can increase cell migration and suppress the synthesis of type I collagen in HSFs 41.

Myopic aqueous humor has variations in miRNA expression, and these molecules regulate ocular signaling pathways.

##### miRNAs associated with myopia in the vitreous humor

Jiang *et al.* discovered a significant decrease in miR-204-5p in vitreous humor (VH) from HM patients. Since the miR-204-5p-TXNIP axis directly targets a thioredoxin-interacting protein (TXNIP) to prevent proliferation, migration, invasion, and apoptosis and reduces reactive oxygen species (ROS) accumulation, it may be involved in regulating retinal cell activity and oxidative damage 42. You *et al.* recovered high-purity exosomes from VH tissues from people with pathological myopia (PM) and a non-myopic group. They discovered that inhibiting miR-145 and miR-143 may increase IGF1R levels, trigger the insulin-resistant pathway, and lead to AL growth. miR-145-5p and miR-143-3p may be markers of myopic maculopathy progression 43. Ando *et al.* discovered that the group of macular holes with HM had significantly higher levels of LET-7c and lower levels of miR-200a compared to the group of macular holes alone. LET-7c targets genes, including MAPK and several inflammatory signaling pathways. miR-200a targets genes, among which PI3K/AKT is the most abundant. In the macular holes with high myopia, the levels of the inflammatory cytokines C-C motif chemokine ligand 2 (CCL2), C-X-C motif chemokine ligand 10 (CXCL10), and interferon-gamma (IFNG) in the vitreous are significantly elevated. This suggests that myopia development, tissue reshaping, inflammation, and AL elongation are linked to LET-7c and miR-200a 44.

Changes in miRNA expression in the vitreous of eyes with macular degeneration and high myopia are linked to pathways involved in tissue remodeling and inflammation. The miRNA-based signal pathway axis controls retinal cell behavior and oxidative stress.

##### miRNAs associated with myopia in the retina

**Figure 3:**
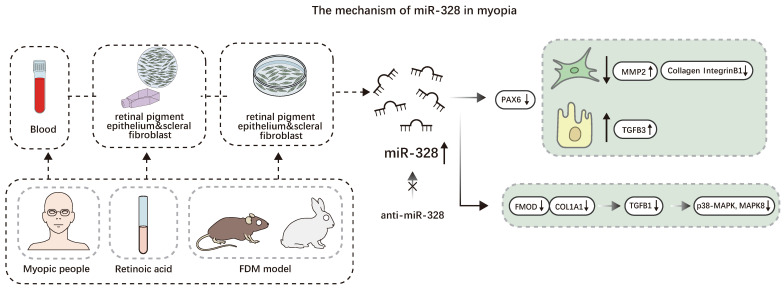
The mechanism of miR-328 in myopia. Elevated expression of miR-328 leads to 1. A decrease in PAX6 results in (1) a decrease in the proliferation of scleral fibroblast, an increase in MMP2, and a decrease in collagen I and integrinB1, and (2) an increase in TGFB3, which causes an increase in the proliferation of retinal pigment epithelium cells. 2. A decrese in FMOD and COL1A1 leads to a decrease in p38-MAPK and MAPK8.

Patients with myopic macular degeneration have elevated levels of hsa-miR-328-3p in the blood, and the RPE layer's optical density decreased 45. The C allele rs662702 of the PAX6 gene increases the risk of myopia, and the expression of miR-328 in the blood cells of myopic patients is higher than that of the average group 46. Chen *et al.* also discovered that the dangerous allele C of the 3'UTR SNP rs644242 did strongly respond to miR-328 and downregulate PAX6 expression. When PAX6 transcription is downregulated, RPE proliferation increases, and HSF proliferation decreases. Type I collagen and integrin subunit beta 1 (ITGB1) are reduced in the sclera, whereas TGFB1 and MMP2 increase. Retinoic acid (RA) increases miRNA-328 expression and inhibits PAX6 expression 47. According to Liang *et al.*, miR-328-3p decreased both protein expression and mRNA dose-dependent manner. Fibromodulin (FMOD) raises p38-MAPK and MAPK8 phosphorylation levels and stimulates TGFB1 expression. Anti-miR-328-3p also inhibits AL elongation 48. Patients with myopia exhibit peripheral blood and retinal upregulation of miR-328, indicating that miR-328 may develop into a biomarker for myopia (Figure 3).

Mei *et al.* detected that miR-466c-5p, miR-466h-5p, miR-466j, miR-468, miR-669e, miR-15a, miR-16-1, and miR-294 were significantly elevated in FDM retinas and whole eye samples. Seven were enriched considerably in transcriptional regulation, axon guidance, and the TGFB1 signaling pathway 49. In addition, a study found that seven elevated miRNAs (miR-101a, miR-6690, miR-466f, miR-291a, miR-465b, miR-696, and miR-18b) were expressed five times greater in the retina of FDM mice than in the sclera. This implies they might play a role in controlling processes specific to the retina. A small group of retinal cells may exhibit miR-145 downregulation, as its expression in the retina is 25.4 times higher than in the sclera 50.

Liu *et al.* found that miR-92b-3p transcription was downregulated in lens-induced myopia (LIM) guinea pigs, resulting in elevated levels of p53 and BTG anti-proliferation factor 2 (BTG2). Activated BTG2 can cause retinal tissue apoptosis, induce DNA damage in guinea pig retinal tissue, promote BCL2 apoptosis regulator (BCL2) expression and apoptosis, and increase the levels of cyclin-dependent kinase 2 (CDK2) and BCL2-associated X (BAX). These effects can lead to decreased retinal thickness, electrophysiological dysfunction, and impaired visual function 51. Myopic retina has higher levels of miR-182-5p, miR-181a-5p, miR-183-5p, miR-9-5p, and miR-96-5p. Myopia and myopia-related retinopathy are caused by overexpression of miR-181a-5p, which can also trigger autophagy, promote RPE cell proliferation, and target N-Sulfoglucosamine Sulfohydrolase (SGSH) in ARPE19 52. In FDM and LIM animal models of the retina, Cui *et al.* discovered that mutations in TEA domain family member 1 (TEAD1) may downregulate miR-671-5p, leading to modifications to cyclic adenosine monophosphate (cAMP) response element binding protein 1 (CREB1) and MAPK1 hub genes that are enriched in extracellular estrogen signaling and visual learning. Additionally, atropine targets MAPK1 and CREB1, key signals in the retinal choroid sclera cascade 53. Liu *et al.* discovered elevated levels of mmu-miR-1936, mmu-miR-673-3p, and mmu-miR-338-5p in the retinas of FDM mice. Their target genes, such as Pax6 and SMAD family member 3 (SMAD3), are primarily enriched in retinal tissue morphogenesis and developmental growth. Through post-transcriptional gene regulation, these three miRNAs may contribute to myopia progression 54.

Overexpression of miR-328 might help in the early detection of myopia. Specific miRNAs are involved in tissue repair and the inflammatory response and are associated with retinal proliferation, autophagy, and apoptosis.

##### miRNAs associated with myopia in the choroid

Li *et al.* discovered that miR-138-5p suppresses the expression of α-smooth muscle actin (α-SMA), hydroxyproline (HYP), interleukin 1 beta (IL1B), TNF, TGFB1, and type I collagen by blocking the HIF1A signaling pathway. It successfully reduced the experimental myopic guinea pigs' axial length and refractive power, improved their choroidal fibrosis, and inhibited the progression of myopia 55. According to Liu *et al.*, peak expression of miR-21 in laser-induced CNV in FMD guinea pigs occurs on day 7, earlier than the expression of HIF1A and VEGF (day 14) and the peak development of CNV (day 21). miR-21 can promote the development of HM guinea pigs' CNV and is well correlated with the HIF1A-VEGF signaling pathway 56. Current research on choroidal miRNAs mainly focuses on choroidal fibrosis and CNV formation.

In summary, miR-138-5p significantly suppresses choroidal fibrosis, while miR-21 can interfere with the establishment of CNV.

##### miRNAs associated with myopia in the sclera

Guo *et al.* found 27 differently expressed miRNAs in the sclerotic membranes of LIM guinea pigs. The primary signaling pathways were closely related to peroxisome proliferator-activated receptor alpha (PPARA), TGFB1, pyruvate, and propionic acid metabolism 57.

Zhang *et al.* found that transfection with miR-29a analogs dramatically increased MMP2 mRNA levels in RPE and scleral fibroblasts (SF), according to Matrix metalloproteinases (MMPs) hydrolyze proteins in the extracellular matrix (ECM). Targeting miR-29a may be beneficial for preventing and treating myopia 58. Yang *et al.* discovered that in SF, elevated miR-29a expression suppresses the expression of type I collagen, MMP2, phosphoinositide, phosphatase, and tensin homolog (PTEN). Although MMP2 downregulation leads to increased type I collagen, miR-29a may more effectively inhibit type I collagen under the action of the gene expression regulatory complex, ultimately leading to a decrease in type I collagen 59. Tang *et al.* found that miR-29a in HSFs inhibits the expression of serpin family H member 1 (SERPINH1), the SMAD3 pathway, COL1A1, and BCL2, upregulates the expression of BAX, reduces the expression level of collagen in HSFs, inhibits the proliferation of HSFs, and increases the apoptosis rate of HSFs 60. In guinea pig myopia models' sclera, Wang *et al.* found an increase in the miR-29 cluster (a, b, and c) in the sclera of a guinea pig myopia model, leading to downregulation of COL1A1 expression and overexpression of MMP2. Genipin can counteract this process by lowering miR-29 expression 61.

Similarly, the G allele at the miR-29a SNP rs157907 locus reduces the incidence of HM, according to a clinical study by Xie *et al.* 62. Jiang *et al.* collected the expression profiles of myopia-related miRNAs (miR-328, miR-184, miR-29a, and miR-7i) and fetal sclera miRNAs (miR-328, miR-103, miR-98, miR-107, miR-29b, miR-LET-7, and miR-214) to provide more accurate prognosis, diagnosis, and response prediction for possible myopia treatment 63.

**Figure 4:**
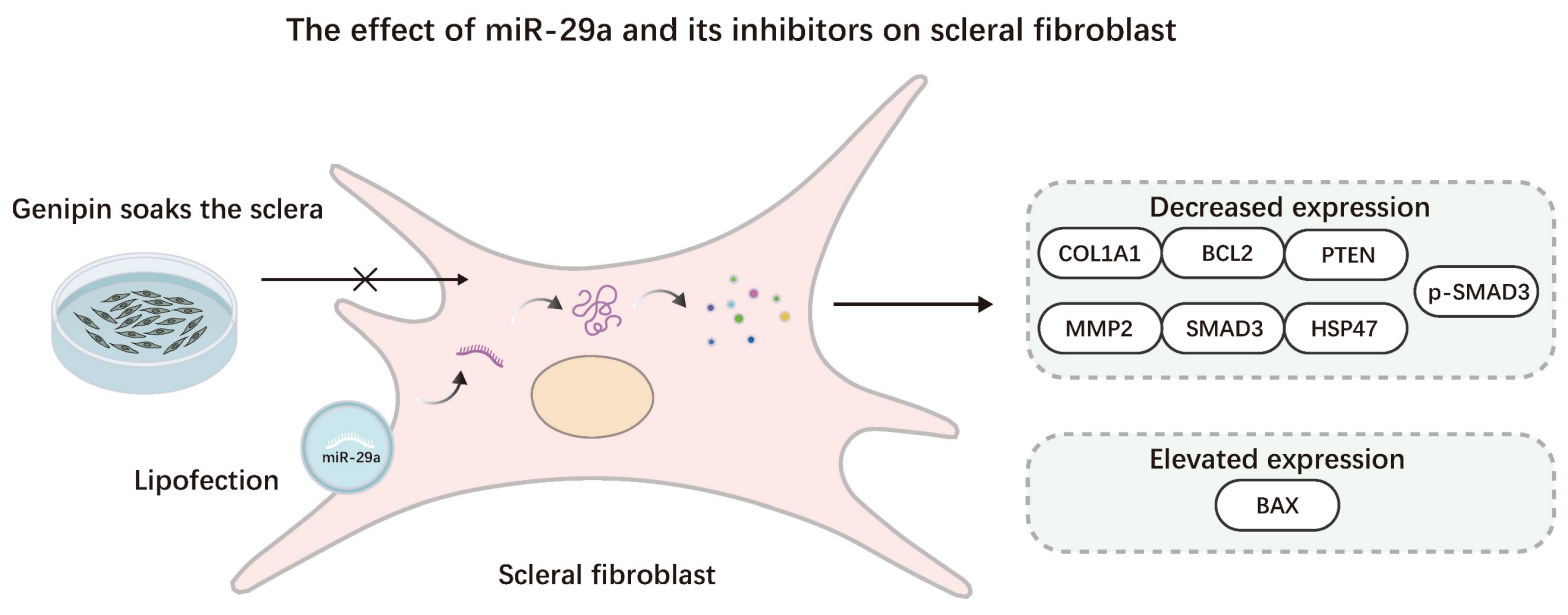
The effect of miR-29a and its antagonists on Scleral fibroblast. Myopia was promoted using the lipofection method to transfect SFs with miR-29a, resulting in elevated BAX expression and reduced COL1A1, BCL2, PTEN, MMP2, SMAD3, HSP47, and p-SMAD3 expression. This process can be reversed by using Genipin.

SF, AH, and blood of myopic patients all contain miR-29a, whose primary function is to prevent the formation of type I collagen from forming in SF, thereby promoting scleral remodeling. This implies that miR-29a may be a potential biomarker for myopia and a target for future myopia treatments. However, the expression trajectory of MMP2 in HSF transfected with miR-29 was different from that in myopic guinea pigs. It is necessary to consider whether species differences exist or whether the cells in the *in vitro* cell experiment are from myopic eyes (Figure 4).

A study found significant variations in the levels of miRNAs (miR-16-2, LET-7a) and mRNAs (peripherin 2 (PRPH2), G protein subunit alpha transducin 1 (GNAT1), and sperm motility kinase 4A (SMOK4A)) in the sclera of FDM mice. G proteins, phototransduction, calcium ions, cytoskeletal protein binding, intermediate filament organization, and stimulus detection are important ontologies of gene overexpression. This implies that myopia is linked to differences in the expression of sclera miRNAs, which controls eye growth 64. Before going to bed at night, atropine eye drops can decrease melatonin breakdown in the SF, down-regulate miR-2682-5p, and target the voltage-gated potassium channel J subfamily member 5 (KCNJ5) and prolactin receptor (PRLR), which reduces eye growth and scleral remodeling 65. Zhang *et al.* reported that in LIM guinea pigs, the miR-15b-5p/miR-379-3p/IGF1R/PTEN/forkhead box (FOXO)/cyclin-dependent kinase inhibitor 1B (CDKN1B) axis can block the G1 cell cycle, induce apoptosis, affect the process of scleral fibrosis, lead to scleral reshaping, and aggravate the development of myopia 66.

Ren *et al.* discovered that the serum of PM patients had high laminin subunit alpha 4 (LAMA4) and low expression of miR-150-5p. LAMA4 activates the p38-MAPK signaling pathway, and miR-150-5p expression was downregulated due to the hypoxia-cultured HSF increasing HIF1A in the promoter region of miR-150-5p. As a result, MMP2 decreased, COL1A1, TIMP metallopeptidase inhibitor 2 (TIMP2), and α-SMA increased, which inhibit HSF ECM degradation and induce PM 67. In the FMD animal model, MMP2 expression is elevated, TIMP and type I collagen expression are reduced 68,69, and sclera ECM degradation is accelerated, leading to ECM remodeling. The results of Ren's study run counter to common sense. Factors such as species differences, differences between *in vitro* and *in vivo* experiments (HSFs are not derived from myopic eyes), and tissue differences should be considered.

Bioinformatics can also be used to search for differentially expressed miRNAs. Tanaka *et al.* found that individual ocular tissues of LIM mice, including the sclera, cornea, retina, iris, lens, and choroid, showed significant overlapping changes in miRNA expression 70. Hsa-miR-17-5p is one of the miRNAs with the most crucial number of gene links, according to Xiao *et al.* Myopia-specific miRNAs control eye development and DA biological processes 71. miR-411-5p, miR-376c-3p, miR-155-5p, miR-132-3p, and miR-543 were downregulated in animal myopia samples and upregulated in human myopia samples. To find biomarkers of human myopia, animal models alone are not enough 72. miRNA expression in human and animal models of myopia is different, and animal experiments combined with cell experiments or clinical trials may be necessary.

Lastly, miRNAs are unrelated to myopia. Two new heterozygous miR-184 alternative variants were found in two cases of isolated keratoconus. The miR-184 stem-loop rs41280052 was not substantially linked to keratoconus, and the axial myopia group had no miR-184 mutations 73.

The sclera of myopic eyes contains differentially expressed miRNAs, including miR-29 and miR-328, which control collagen, ECM proteins, and associated signal pathways in various tissues. These miRNAs are also involved in the remodeling and degradation of the scleral ECM and affect eye growth. They expected to be a targete for myopia treatment. However, studies using FDM and LIM models show that the expression patterns of myopia-related miRNAs in human and animal models are different. This suggests that caution should be exercised when comparing studies conducted in other species.

In conclusion, miRNAs are predicted to develop into myopia biomarkers since they exhibit differential expression in different tissues of myopic individuals. Numerous miRNAs control cell migration, proliferation, and apoptosis and are associated with eye development, tissue remodeling, and fibrosis. By influencing the metabolism of collagen and ECM, some miRNAs can control scleral remodeling and the length of the ocular axis. They may be used as therapeutic targets for future myopia treatment. Most miRNA research, however, has been conducted in animal models (such as FDM and LIM animal models), and the patterns of miRNA expression in these models differ from those in humans. It takes time and effort to extrapolate animal research accurately results to humans. Moreover, miRNAs play distinct roles in various tissues and experimental paradigms. More accurate experimental design and analysis are needed to investigate the tissue specificity of miRNAs and their mechanism of action in myopia. Lastly, one miRNA can affect multiple target genes and signal pathways due to its multi-target effect. Future studies must identify more precise targets, as this widespread regulatory action may have negative implications.

#### 3.2.2. Long non-coding RNAs

Long non-coding RNAs (lncRNAs) are generally considered to be ncRNA molecules longer than 200 nucleotides with a 3' poly(A) tail and a 5' 7-methylguanosine cap 74. Many aspects of cell development, differentiation, and other physiological processes are regulated by lncRNAs 75.

Wang *et al.* suggested that the lncRNA-XR_002792574.1/miR-760-3p/adenylate cyclase 1 (ADCY1) axis may negatively affect RGCs in FDM guinea pigs by inhibiting the Apelin and cyclic guanosine monophosphate (cGMP)/protein kinase cGMP-dependent 1 (PKG) signaling pathways. The ERK-MMP2 pathway may also contribute to myopic scleral remodeling 76. Wu *et al.* discovered that the lncRNAs in the ciliary body of LIM guinea pigs are mainly enriched in the complement, cytokine-cytokine receptor interactions, coagulation cascade, and RAP1 GTPase activating protein (RAP1GAP) signaling pathways. Three hub genes—phosphoinositide-3-kinase regulatory subunit 1 (PIK3R1), ITGB1, and catenin beta 1 (CTNNB1)—are involved in the RAP1GAP signaling pathway. Retinal photoreceptors primarily express two key genes: G protein-coupled receptor kinase 1 (GRK1) and phosphodiesterase 6A (PDE6A). They improve the ciliary body's visual perception 77. Li *et al.* identified 655 lncRNAs differently expressed in FDM retinas. These lncRNAs were primarily enriched in retinol metabolism, cytokine-cytokine receptor interactions, cellular activities, and rhythmic processes. lncRNA Gm35369 was mainly expressed in horizontal cells and RGCs 78. Geng *et al.* found aberrant expression of the posterior pole lncRNA in the eyes of FDM and LIM guinea pigs compared to the healthy group. This expression is primarily enriched in cellular components (structural components of ECM), molecular functions (kinase activity, metabolism, and growth), and pathways (such as ECM receptor interaction, glycosaminoglycan degradation, and mucin-type O-glycan biosynthesis) 79. According to the idea of molecular structural effects proposed by Wang *et al.*, lncRNAs fold into complex spatial patterns that attract transcription factor (TF) targets for scaffold binding. Base mutations affecting key components and variable splicing regions have been discovered in transcription factor binding sites (TFBSs) of myopia-associated lncRNA transcripts. These mutations caused substantial changes in molecular conformation and mediate the development and progression of myopia 80. The research on myopia-related lncRNAs mainly involves sequencing animal models' eye tissues (guinea pigs and mice) to find target genes, enriched networks, and specifically expressed lncRNAs.

In short, lncRNAs affect signal transduction, cell proliferation, and remodeling in different tissues in myopia. Their complex spatial structure allows them to participate in multiple physiological pathways. In addition to providing new molecular indicators for myopia development, lncRNAs may also become targets for intervention in tissue damage and structural remodeling linked to myopia. However, target identification is challenging due to the complicated three-dimensional structure of lncRNA and their multi-target characteristics, which may lead to adverse reactions. Furthermore, the stability and distribution system of lncRNAs are still problematic. Future studies must expand the structural and functional analysis of lncRNAs to support their clinical application and the development of precise regulatory techniques.

#### 3.2.3. Circular RNAs

Exon circular RNAs (circRNAs) are covalently closed endogenous RNAs produced by the anti-splicing process. Their functions include protein recruiters, decoys, sponges, scaffolds, and miRNA. They can also encode various proteins, from tiny peptides to proteins, and act as translation templates in pathological processes 81,82.

Zhang *et al.* found that has-circ-Nbea and has-circ-Pank1 promote AL growth in HM patients; the target genes are mainly concentrated in MTOR, insulin, cAMP, vascular endothelial growth factor, and other signaling pathways. The sclera of the FDM mouse model contains the circNbea/miR-204-5p/ inositol 1,4,5-trisphosphate receptor type 1 (ITPR1) and circPank1/miR-145-5p/ NRAS proto-oncogene (NRAS) axes. These axes could be targets for myopia treatment or biomarkers for myopia progression 83. Ma *et al.* discovered that compared with ARC patients, high myopia cataract (HMC) patients have increased tropomyosin 1 (TPM1) expression by circ-AFF1 through sponge miRNA760. It also controls apoptosis, proliferation, and migration of lens epithelial cells (LECs) 84. Current research on circRNAs focuses on RNA sequencing of VH and lens epithelium samples from myopic patients to map circRNA-related networks and profiles.

Through particular signaling pathways, circRNAs can control the sclera and promote the growth of AL. They may also be involved in pathological changes of the eye's lens and can prevent the growth and death of LECs. By interfering with the pathway regulated by circRNA, it should be possible to regulate the myopic lesion tissue precisely. However, due to the complex regulation mechanism of circRNAs, learning more about their specific roles as protein and miRNA sponges is essential.

#### 3.2.4. Short interfering RNAs

Dicer enzymes cleave non-coding double-stranded RNA (dsRNA) into short interfering RNAs (siRNAs) 85. RNA interference (RNAi) is an evolutionarily conserved mechanism for post-transcriptional gene silencing 86. siRNAs control gene expression via RNAi. They can also be used as a possible therapeutic agent. The local distribution of naked siRNA in two tissues, the lung and the eye, shows promise 87.

siRNAs can be used to knockout genes in the sclera that are associated with myopia. When CHRM2 in the sclera of mice is knocked out using siRNA, the levels of type I collagen increase, and type V collagen decreases, the growth of SF is reduced, and mice without CHRM2 were less likely to develop LIM than wild-type mice 88. The use of transglutaminase 2 (TGM2) specific siRNA in HSFs slowed the prolongation of AL. Drugs that block CHRM2 also slowed myopia progression in mice, and this effect was accompanied by a decrease in TGM2 expression 89.

Furthermore, Zhao *et al.* discovered that COL1A1 is downregulated in HSF when PDE4B is knocked down by siRNA, indicating that PDE4B may be a new gene susceptible to HM 90. Myofibroblast development is controlled by the ras homolog family member A (RHOA) / rho-associated coiled-coil containing protein kinase 2 (ROCK2), a critical mechanotransduction pathway. Elevated α-SMA expression is due to upregulated RhoA expression leading to activation of ROCK2. Mechanical strain cannot enhance the production of ROCK2, myocardin-related transcription factor A (MRTFA), and SRF if siRNA is used to block RhoA 91. Li *et al.* found that the LIM group transfected with hepatocyte growth factor (HGF) siRNA had significantly lower MMP2 protein expression in SF than the normal group, indicating that HGF is an upstream mediator of MMP2 in guinea pigs SF 92.

siRNAs can also knock out myopia-related genes in the retina and lens. Dopamine receptor D2 (DRD2) protein expression increased in human RPE when ADORA2A was knocked down using siRNA, suggesting that ADORA2A and DRD2 heterodimers are present. To limit myopia development, 7-methylxanthine (7-MX) can increase the expression of DRD2, pERK1/2, and ERK1/2 while inhibiting the expression of ADORA2A 93. HM is associated with endoplasmic reticulum stress-induced apoptosis of LECs, which causes cataracts. Apoptosis of human LECs is induced by exposing human LEC cells to endoplasmic reticulum stress under UV irradiation through activation of the activating transcription factor 4 (ATF4)-ATF3 -DNA damage-inducible transcript 3 (DDIT3) pathway. Downregulating ATF4, ATF3, and DDIT3 by siRNA can block KLF transcription factor 6 (KLF6)-induced apoptosis 94.

In other words, siRNA can knock out particular genes to confirm their function. It is a precise treatment technique that can silence genes linked to myopia. However, enhancing siRNA's specificity and targeting while minimizing off-target effects is a key therapeutic issue. The stability of siRNA in clinical applications needs to be further optimized.

#### 3.2.5. Ribosomal RNAs

Ribosomal RNA (rRNA) accounts for about 80% of cellular RNA 95. In all known organisms, rRNA is essential for protein synthesis and maintaining cellular function 96. In bacteria, the structure of 16S rRNA has evolved into a highly conserved and diverse part with a medium-length gene sequence. This biomarker sequence is ideal 97.

In myopia, 16s rRNA is mainly used to reveal the potential relationship between the microbial community (primarily intestinal microorganisms) and myopia and confirm the existence of the gut-eye axis.

Fecal samples from adolescents sequenced with 16S RNA showed that the microbial communities in the myopia group were different from those in the control group in composition and abundance. The Firmicutes and Actinobacteria phyla had different relative abundances and absolute quantities, suggesting that the characteristics of the intestinal flora can significantly distinguish myopia from healthy controls 98. According to 16S rRNA analysis, the intestinal microbiota of LIM mice—myopic mice with impaired intestinal barrier function—showed a much lower relative abundance of the Firmicutes phylum and a much higher relative abundance of the Actinobacteria phylum 99. Wu *et al.* found through 16S rRNA gene sequencing that Ruminococcus_albus is a dominant and abundant bacterium in the intestines of FDM guinea pigs and is positively correlated with the levels of ten metabolites such as L-glutamic acid, as well as with vasoactive intestinal peptide (VIP) and lipopolysaccharide (LPS) 100. The amount of the intestinal microbial metabolite indole-3-acetic acid (3-IAA) in the plasma of HM patients and the amount of beneficial intestinal bacteria were significantly reduced using 16S rRNA gene sequencing. When HM mice received a fecal microbiota transplant from a healthy donor, 3-IAA plasma levels increased, and HM progression slowed. The expression of COL1A1 in the sclera is maintained through Sp1 transcription factor (SP1)-dependent transcriptional activation 101.

The researchers also performed 16s rRNA sequencing on conjunctival sac swab specimens. They found that patients with HM had higher scores on the Ocular Surface Disease Index (OSDI), an imbalance in the bacterial microbiome in the conjunctival sac. Proteus, Actinomyces, and Acinetobacter may be associated with HM-related ocular surface inflammation 102.

In conclusion, 16S rRNA sequencing showed that the intestinal and ocular surface microbiomes of myopic eyes are significantly different from those of healthy eyes, thus confirming the existence of the gut-eye axis. Therefore, 16S rRNA sequencing can help with the early myopia diagnosis. By controlling the intestinal microbiota, fecal microbiota transplantation (FMT) may slow the progression of HM. Yet, the microbiomes of myopic patients vary greatly, and the long-term effectiveness and safety of FMT need to be confirmed by further research.

#### 3.2.6. Transfer RNAs

A small ribonucleic acid molecule known as transfer RNA (tRNA) carries and transports amino acids. Under the direction of mRNA, its main job is to transport amino acids to the ribosome for protein synthesis.

Studies on the connection between tRNA and myopia are scarce. In the growing fetal brain, glutaminyl-tRNA synthetase (QARS) is extensively expressed. It is hypothesized that the catalytic domain of the QARS gene contains a homozygous missense mutation (V476I). It then causes glutamine-tRNA synthetase deficiency, which induces a novel recessive disease, including intellectual disability, microcephaly, severe linear growth retardation, HM, syndactyly, and distinctive facial features 103.

Briefly, QARS mutations can be used as genetic indicators of high myopia and provide clues for the early detection of rare myopia-related diseases. However, more research is still needed to determine their mode of action.

When ncRNA-related research is summarized, these RNA molecules have multiple functions in myopia's genetic control, signal regulation, and microbiomics. This information is crucial for understanding myopia's molecular mechanism and guiding the development of prospective therapeutic approaches. However, the clinical translation of the research results may be hampered by differences in RNA expression between human myopia and animal models. Determining the differences in RNA expression between humans and other species is crucial. Second, because ncRNAs are widely expressed in various tissues, it is challenging to ensure therapeutic specificity and prevent side effects.

### 3.3 Histone modification

Histones and their core octamer complexes form the basic structural unit of eukaryotic chromosomes 104. The ribosomal core particles are formed by wrapping the DNA of each cell around the histone octamer. Numerous residues at these termini are susceptible to post-translational modification, which affects all DNA-based functions 105.

#### 3.3.1 Histone methylation

Lysine methylation marks different sites on the tail and globular domains of histones, and a precise balance is achieved through the action of methyltransferases (“writers”) and demethylases (“erasers”); in addition, how different effector proteins (“readers”) recognize specific methyl lysines depends on the adjacent amino acid sequence and methylation status. H3K27me3 is a classic histone modification site 106,107.

Cui's team found that heat shock transcription factor 4(HSF4) recruits the methyltransferase enhancer of zeste 2 polycomb repressive complex 2 subunits (EZH2), increased H3K27me3 levels in the proximal promoter region of p21cip1, inhibit p21cip1 expression in mouse embryonic fibroblasts (MEFs), reduced UVB-induced senescence of LECs, and suggested that HSF4 may have an anti-aging effect during lens development 108. Methyltransferases that regulate G9a, H3K27me3, EZH2, and H3K9me2 in the mouse retina are enriched during active retinal development in the embryo. RGCs exhibit H3K9me2 during their early development. These histone marks and the related methyltransferase mediate the epigenetic regulation of essential cell lineage genes throughout adult retinal cell plasticity, axonal regeneration, retinal cell capacity, retinal development, retinal cell survival, and retinal cancer 109. Sumiko *et al.* learned that the methylation levels of histones H3K27 and H3K4, which are essential for retinal differentiation and proliferation, as well as the acetylation levels of histone H3 at specific gene loci, undergo significant changes during retinal development. Progenitor cell-specific gene expression is attenuated by low amounts of H3K4me3 and high amounts of H3K27me3 110.

Genetic regulation of ocular tissue throughout development can be better understood by considering the effects of histone methylation on retinal development and lens cell aging.

#### 3.3.2 Histone acetylation

Acetylation is a common and degradable post-translational protein modification catalyzed by two types of enzymes: lysine deacetylases (KDACs) remove the acetyl group, and lysine acetyltransferases (KATs) transfer the acetyl group to the lysine residue 111.

**Figure 5:**
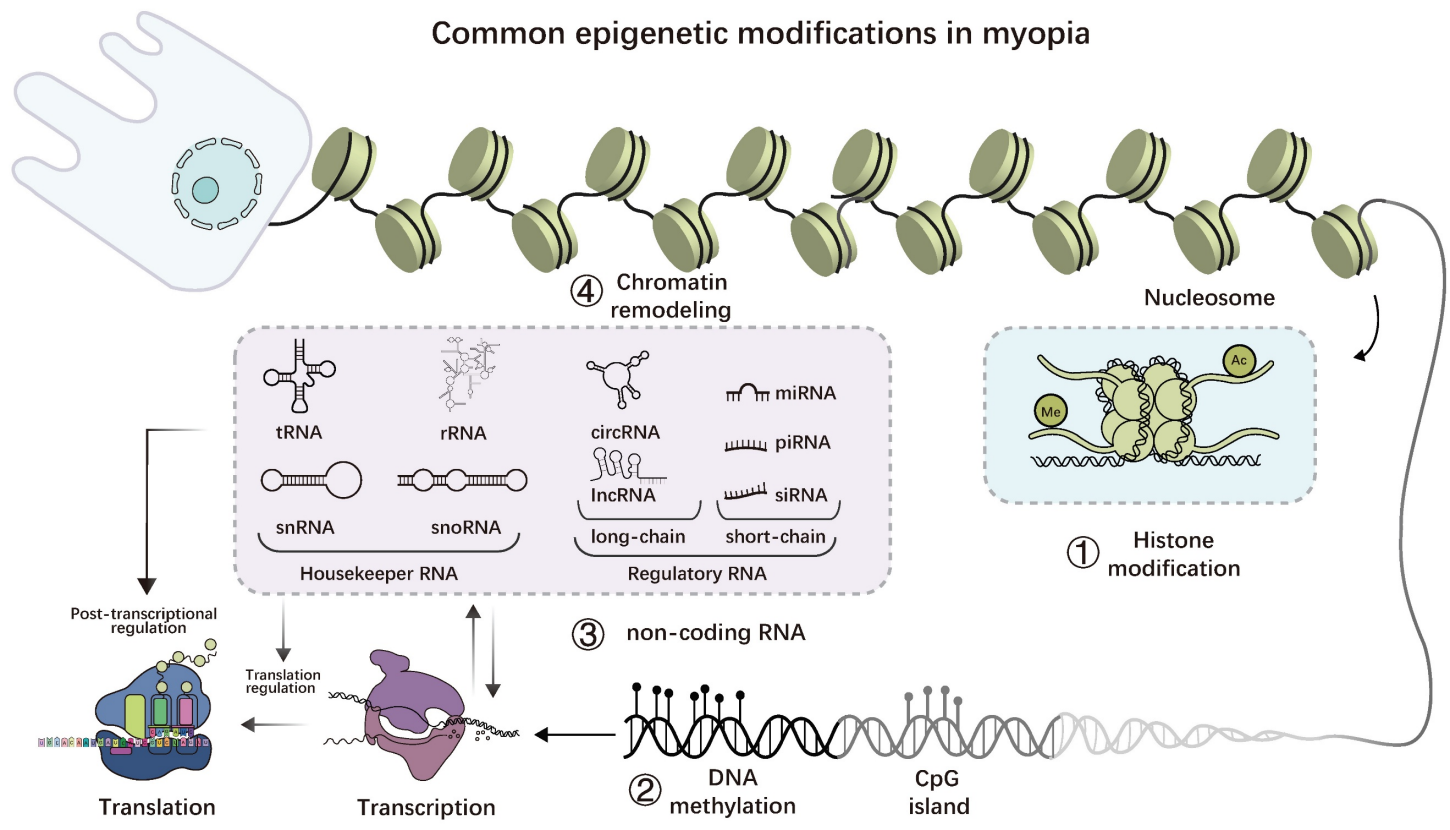
Common epigenetic modifications in myopia. 1. histone modification is a reversible covalent modification that mainly occurs at the tail of histones; 2. DNA methylation occurs on CpG islands, and the methyl group is acquired via covalent binding; 3. ncRNAs, including housekeeper and regulator types; 4. RNA methylation is a reversible post-transcriptional modification that primarily occurs on mRNA but is also found in non-coding RNAs, with N6-methyladenosine (m6A) being the most common form; 5. Numerous enzymes, including the SWI/SNF complex, are involved in chromatin remodeling. By interacting with one another, the four common epigenetic changes mentioned above can also impact gene expression.

One study found that elevated level of glycolytic activity in the optic nerve sheath region of mouse embryos. Eye development was inhibited by removing the activity of the lactate dehydrogenase A (LDHA) gene and the solute carrier family 2 member 1 (SLC2A1) in developing retinal progenitor cells. Histone H3 acetylation is epigenetically modified by histone deacetylase (HDAC) operation, which regulates the lactate-mediated key field-specific transcription factors 112. According to Park *et al.*, the expression levels of HDAC2 and HDAC3 in limbal conjunctiva (LC) and peripapillary sclera (PPS) ECM of Koreans are lower than Caucasians ECM, the expression levels of acetylated histone H3, the elastic fiber components (fibrillin 1 and elastin) and lysine oxidase-like protein (LOXL2) are higher than those in Caucasians, indicating that the histone acetylation status of elastic fiber component and LOXL2 promoter region differs in LC and PPS of other ethnic groups 113. HM is associated with five non-coding potassium voltage-gated channel Q member 5 (KCNQ5). SNPs rs7744813 and rs9342979 are located at or near the DNase I hypersensitive sites and histone marks of H3K27ac and H3K4me1. This demonstrates that KCNQ5 is a risk factor for HM and that KCNQ5 gene polymorphisms contribute to genetic predisposition to HM 114. Histone acetylation is associated with eye development and HM (Figure 5).

The reversibility of lysine acetylation makes it possible to intervene in myopia development. Racial differences in histone acetylation reveal its function in the ocular architecture of different races and help develop tailored treatment plans for specific groups.

Changes in histone make new targets for myopia treatment possible. They can be employed as tools to control gene expression in the eye because of their reversibility, which will help to understand the pathophysiology and genetic basis of eye disorders. Racial differences in histone modifications make treatment for specific populations possible. However, histone modification involves multiple enzymes and regulatory pathways, and the regulatory mechanism is complex. Accurately controlling these processes takes time and effort. Finding a more unified treatment is difficult since the effects of histone modification fluctuate widely between races and individuals.

### 3.4 RNA methylation

RNA methylation is a modification process catalyzed by RNA methyltransferases that incorporates methyl groups into RNA bases using s -adenosyl- l-methionine (SAMe) as a donor 115. These include n6-methyladenosine (m6A), n1-methyladenosine (m1A), 5-methylcytosine (m5C), and 7-methylguanosine (m7G) 116.

Wen *et al.* found that upregulation of the m6a methylase methyltransferase-like 14 (METTL14) and downregulation of the demethylase obesity-associated protein (FTO), alkB homolog 5 (ALKBH5), catalyzed the hypermethylation of the m6a methylase-like protein 1 (CHI3L1) and its encoding protein, YKL-40, in the anterior capsule of the lens in patients with nuclear cataracts with high myopia, and contributes to the pathological state of high myopia by regulating the composition of the extracellular matrix. The remaining target genes also include collagen alpha-3(VI) chain (COL6A3) and peroxidasin homolog (PXDN) 117. Xue *et al.* found that forkhead box M1 (FOXM1) increased the m6A methylation level of apolipoprotein A1 (APOA1) by inhibiting methyltransferase-like 3 (METTL3) transcription. APOA1 mRNA stability and transcription were enhanced by decreasing YTHN6- methyladenosine RNA binding protein F2 (YTHDF2)-recognized m6A-methylated transcripts 118. Shan's team found that elevated levels of FTO in neovascularized corneas and endothelial cells of CNV decreased m6A methylation levels of focal adhesion kinase (FAK), leading to reduced RNA stability and increased RNA decay via YTHDF2, resulting in abnormal endothelial cell function and pathological corneal angiogenesis 119. Liu *et al.* found that tRF-22 of the transfer RNA-derived fragments (tRFs) family was decreased in the choroid of C57BL/6 J mice with FDM and interacted with METTL3 to block Axin1 and AT-rich interaction domain 1B (ARID1B) methylation of m6A in mRNA transcripts leads to increased expression of Axin1 and Arid1b, resulting in their attenuated inhibition of Wnt signaling and promotion of choroidal neovascularization 120.

m6A plays a key role in myopia and ocular diseases and could provide new molecular targets for future therapies, especially in regulating gene expression and neovascularization. Although studies have revealed the role of m6A in myopia, the molecular mechanisms involved are still not completely clear, and m6A modifications and their roles still need to be studied. These studies are based on animal models and cellular experiments, and how to effectively translate them into clinical therapeutic approaches still faces excellent technical and practical challenges.

To date, we have summarized the research results on epigenetic modifications in myopia. Current research mainly focuses on ncRNAs, and there are few studies on histone modification, RNA methylation, and chromatin remodeling. In addition, relatively few studies have been conducted on the interactions between different types of epigenetic modifications. Myopia involves multiple epigenetic changes, and other types do not exist in isolation; they form a complex gene regulation network. More critical discoveries may come from connections between various epigenetic changes rather than changes in epigenetic modifications themselves.

## 4. Conclusion

Recent advancements in the study of epigenetic modifications have clarified the complex interactions between genetic susceptibility and environmental factors in myopia development. It is clear that epigenetic processes, including non-coding RNAs (ncRNAs), DNA methylation, histone modifications, and RNA methylation, play a crucial role in regulating gene expression patterns associated with refractive error and ocular development. Gene mutations are not the only factors determining myopia; they involve heritable but reversible epigenetic changes influenced by environmental factors.

DNA methylation patterns of key genes, such as those involved in eye growth and signaling pathways, are associated with myopia development. Furthermore, histone modifications can impact chromatin structure and gene accessibility, thereby affecting the regulation of ocular growth. In myopia, ncRNAs, particularly microRNAs, act as essential regulators of gene expression, providing a possible molecular mechanism for further control of eye elongation and growth. m6A RNA methylation affects myopia development by regulating gene expression and neovascularization and may be involved in key processes such as visual axis lengthening.

Future research should elucidate how to reverse or prevent these epigenetic changes to develop potential therapeutic interventions. Specifically, studies could explore epigenetic therapies, such as DNA demethylation agents or miRNA-based treatments, to avoid or mitigate myopia progression. Additionally, combining genomic, transcriptomic, and epigenetics data will provide a more comprehensive understanding of the molecular causes of myopia and enable the development of personalized approaches to prevention and treatment.

Overall, studying epigenetic modifications provides a promising approach to addressing the rising global prevalence of myopia. Understanding how environmental factors affect gene expression through epigenetic mechanisms can help develop effective strategies for early diagnosis, prevention, and individualized treatment of myopia.

## Abbreviations

3-IAA: intestinal microbial metabolite indole-3-acetic acid
7-MX: 7-methylxanthine
ADCY1: adenylate cyclase 1
ADCY3: adenylate cyclase 3
ADORA2A: adenosine A2a receptor
AH: aqueous humor
AL: axial length
AP1B1: adaptor-related protein complex 1 subunit beta 1
ARC: age-related cataracts
ATF4: activating transcription factor 4
BAX: BCL2-associated X
BCL2: BCL2 apoptosis regulator
BTG2: BTG anti-proliferation factor 2
cAMP: cyclic adenosine monophosphate
CCL2: C-C motif chemokine ligand 2
CDK2: cyclin-dependent kinase 2
CDKN1B: cyclin-dependent kinase inhibitor 1B
cGMP: cyclic guanosine monophosphate
CHRM2: cholinergic receptor muscarinic 2
circRNAs: circular RNAs
CNGB3: cyclic nucleotide-gated channel subunit beta 3
CNV: choroidal neovascularization
COL1A1: collagen type I alpha 1 chain
CpG islands: cytosine-phosphate-guanine sites
CREB1: cAMP response element binding protein 1
CRYAA: crystallin alpha A
CTNNB1: catenin beta 1
CXCL10: C-X-C motif chemokine ligand 10
DA: Dopamine
DDIT3: DNA damage-inducible transcript 3
DNMTs: DNA methyltransferases
DRD2: dopamine receptor D2
dsRNA: double-stranded RNA
ECM: extracellular matrix
EGR1: early growth response 1
EZH2: enhancer of zeste 2 polycomb repressive complex 2 subunits
FDM: form-deprivation myopia
FGF2: fibroblast growth factor 2
FMOD: fibromodulin
FOS: AP-1 transcription factor subunit
FOXO: forkhead box
GNAT1: G protein subunit alpha transducin 1
GRB2: growth factor receptor binding protein 2
GRK1: G protein-coupled receptor kinase 1
GSTP1: glutathione S-transferase pi 1
HDAC: histone deacetylase
HGF: hepatocyte growth factor
HIF1A: hypoxia-inducible factor 1 subunit alpha
HM: high myopia
HMC: high myopia cataract
HOXA9: homeobox A9
HSF4: heat shock transcription factor 4
HSFs: human scleral fibroblasts
HYP: hydroxyproline
IFN1: type I interferon
IFNG: interferon-gamma
IGF1: insulin-like growth factor 1
IGF1R: insulin-like growth factor 1 receptor
IL1B: interleukin 1 beta
ITGB1: integrin subunit beta 1
ITPR1: inositol 1,4,5-trisphosphate receptor type 1
KATs: lysine acetyltransferases
KCNJ5: voltage-gated potassium channel J subfamily member 5
KCNQ5: potassium voltage-gated channel subfamily Q member 5
KDACs: lysine deacetylases
KLF6: KLF transcription factor 6
LAMA4: laminin subunit alpha 4
LC: limbal conjunctiva
LDHA: lactate dehydrogenase A
LECs: lens epithelial cells
LIM: lens-induced myopia
LINE-1: long interspersed nucleotide element 1
lncRNAs: long non-coding RNAs
LOXL2: lysine oxidase-like protein
LPS: lipopolysaccharide
LTA: lymphotoxin alpha
LUM: lumican
MAPK: mitogen-activated protein kinase
MEFs: mouse embryonic fibroblasts
miRNAs: micro RNAs
MMP2: matrix metalloproteinase 2
MMPs: matrix metalloproteinases
mRNA: messenger RNA
MRTFA: myocardin-related transcription factor A
MTOR: mechanistic target of rapamycin kinase
MX1: MX dynamin-like GTPase 1
NAB2: NGFI-A binding protein 2
ncRNAs: non-coding RNAs
NRAS: NRAS proto-oncogene‌
OSDI: Ocular Surface Disease Index
PAX6: paired box 6
PCDHA10: protocadherin alpha 10
PDE4B: phosphodiesterase 4B
PDE6A: phosphodiesterase 6A
PDGFRA: platelet-derived growth factor receptor alpha
PI3K/AKT: phosphatidylinositol 3 kinase/protein kinase B
PIK3R1: phosphoinositide-3-kinase regulatory subunit 1
piRNA: piwi-associated RNA
PKG: protein kinase cGMP-dependent 1
PM: pathological myopia
PPARA: peroxisome proliferator-activated receptor alpha
PPS: peripapillary sclera
PRLR: prolactin receptor
PRPH2: peripherin 2
PSEN1: presenilin 1
PTEN: phosphatase and tensin homolog
QARS: glutaminyl-tRNA synthetase
RA: Retinoic acid
RAP1GAP: RAP1 GTPase activating protein
RGCs: retinal ganglion cells
RGS5: regulator of G protein signaling 5
RHOA: ras homolog family member A
RISC: RNA-induced silencing complex
RNAi: RNA interference
ROCK2: rho-associated coiled-coil containing protein kinase 2
ROS: reactive oxygen species
RPE: retinal pigment epithelial cells
rRNA: ribosomal RNA
RUNX1: RUNX family transcription factor 2
SERPINH1: serpin family H member 1
SF: scleral fibroblasts
SGSH: N-sulfoglucosamine sulfohydrolase
siRNAs: short interfering RNAs
SLC2A1: solute carrier family 2 member 1
SMAD3: SMAD family member 3
SMOK4A: sperm motility kinase 4A
snoRNA: small nucleolar RNA
snRNA: small nuclear RNA
SOCS1: suppressor of cytokine signaling 1
SP1: Sp1 transcription factor
SRF: serum response factor
TEAD1: TEA domain family member 1
TF: transcription factor
TFBSs: transcription factor binding sites
TGFB: transforming growth factor beta
TGM2: transglutaminase 2
TIMP2: TIMP metallopeptidase inhibitor 2
TNF: tumor necrosis factor
TPM1: tropomyosin 1
tRNA: transfer RNA
TXNIP: thioredoxin-interacting protein
TXNRD2: thioredoxin reductase 2
V476I: homozygous missense mutation
VEGFA: vascular endothelial growth factor A
VH: vitreous humor
VIP: vasoactive intestinal peptide
ZNRF3: zinc and ring finger 3
α-SMA: α-smooth muscle actin
